# Case Report : Use of Low Dose Nabilone to Manage BPSD in Alzheimer’s Disease

**DOI:** 10.1192/bjo.2026.11792

**Published:** 2026-06-30

**Authors:** Amulya Koneru, Manhal Zarroug

**Affiliations:** South London and Maudsley NHS Foundation Trust, London, United Kingdom

## Abstract

**Aims:**

Behavioural and psychological symptoms of dementia(BPSD) are very common in people with dementia, including Alzheimer’s disease.The prevalence is very varied,ranging from 25%-75%.There are many treatment options available including pharmacological and non-pharmacological methods.We present a patient with Alzheimer’s disease whose BPSD were successfully managed with the synthetic cannabinoid,Nabilone.

**Methods:**

Mr. KS,a 73-year-old gentleman with multiple physical comorbidities and a family history of dementia,was admitted to the ward with complaints of worsening memory and functioning since a few months.He had severe irritability,agitation,verbal and physical aggression along with suspiciousness towards his family.On assessment, his mood was irritable with delusion of persecution;he was disoriented to time and place and had poor recent memory along with word-finding difficulties.Delirium was ruled out.Cognitive assessment was done using Standardised Mini-Mental State Examination and he scored 13/30.Brain imaging(MRI) showed mild vascular changes and medial temporal lobe atrophy score of 3.A diagnosis of Dementia in Alzheimer’s disease with late onset was made.Clinical Dementia Rating(CDR) Global Score showed 2 which meant moderate dementia and he was started on Memantine.BPSD symptoms continued to be severe in nature.Various non-pharmacological techniques(redirection,ABC method,activity engagement) were tried with minimal effect.Following this,medications such as lorazepam,melatonin,and trazodone were tried.Though there was partial improvement with trazodone,he continued to be severely agitated and aggressive.Hence,following discussion with the team including senior pharmacists,nabilone 500mcg daily was initiated.

**Results:**

Nabilone is a synthetic cannabinoid of delta-9 tetrahydrocannabinol(THC) which has weak partial agonist activity at Cannabinoid-1 and Cannabinoid-2 receptors.The use of nabilone in dementia for BPSD management is an off-licence indication.For this patient,NPI(neuropsychiatric inventory) was done and he scored 51 in frequency and severity score and 21 in distress score,with high scores in the domains of delusions,agitation/aggression,disinhibition,irritability/lability,and sleep and nightmare behaviour disorders.Following introduction of nabilone,his agitation and aggression improved significantly within 2 weeks.After 3 weeks,NPI was repeated and he scored 9 in frequency and severity score and 5 in distress score with improvement in all domains including care-giver distress.There was a trial to decrease his Nabilone to 250 mcg–however his behavioural symptoms worsened so he was put back on 500mcg.He is maintaining well on this dose.

**Conclusion:**

There have been many trials looking into the effectiveness of nabilone in managing non-cognitive symptoms of dementia.In patients who have been tried on conventional treatment regimens and have had poor response,it is an effective option and can be considered in managing BPSD.We have successfully managed this patient with a low dose of nabilone with no significant long-lasting side-effects.

